# The MADS transcription factor CmANR1 positively modulates root system development by directly regulating *CmPIN2* in chrysanthemum

**DOI:** 10.1038/s41438-018-0061-y

**Published:** 2018-10-01

**Authors:** Cui-Hui Sun, Jian-Qiang Yu, Xi Duan, Jia-Hui Wang, Quan-Yan Zhang, Kai-Di Gu, Da-Gang Hu, Cheng-Shu Zheng

**Affiliations:** 1 National Key Laboratory of Crop Biology, College of Horticulture Science and Engineering, Shandong Agricultural University, Tai-An, Shandong 271018 China; 2 Shandong Agricultural and Engineering University, Ji-Nan, Shandong China

## Abstract

Plant root systems are essential for many physiological processes, including water and nutrient absorption. MADS-box transcription factor (TF) genes have been characterized as the important regulators of root development in plants; however, the underlying mechanism is largely unknown, including chrysanthemum. Here, it was found that the overexpression of *CmANR1*, a chrysanthemum MADS-box TF gene, promoted both adventitious root (AR) and lateral root (LR) development in chrysanthemum. Whole transcriptome sequencing analysis revealed a series of differentially expressed unigenes (DEGs) in the roots of *CmANR1-*transgenic chrysanthemum plants compared to wild-type plants. Functional annotation of these DEGs by alignment with Gene Ontology (GO) terms and biochemical pathway Kyoto Encyclopedia of Genes and Genomes (KEGG) enrichment analysis indicated that CmANR1 TF exhibited “DNA binding” and “catalytic” activity, as well as participated in “phytohormone signal transduction”. Both chromatin immunoprecipitation–polymerase chain reaction (ChIP-PCR) and gel electrophoresis mobility shift assays (EMSA) indicated the direct binding of *CmPIN2* to the recognition site CArG-box motif by CmANR1. Finally, a firefly luciferase imaging assay demonstrated the transcriptional activation of *CmPIN2* by CmANR1 in vivo. Overall, our results provide novel insights into the mechanisms of MADS-box TF CmANR1 modulation of both AR and LR development, which occurs by directly regulating auxin transport gene *CmPIN2* in chrysanthemum.

## Crop genetics: The genes that make chrysanthemum roots

Researchers in China have elucidated the genetic components controlling root development and architecture in chrysanthemum. A team at Shandong Agricultural University led by Da-gang Hu showed that the *ANR1* gene promotes root branching as well as the development of additional roots from stem tissue. To identify other genes in the pathway, the team sequenced the RNA of plants with increased *ANR1* expression to identify. They found 7,600 genes regulated by *ANR1*, including genes related to the plant hormone auxin, which is known to regulate root development. Further experiments revealed that *ANR1* directly activates the *PIN2* gene, which plays a role in determining the auxin distribution in the root. These findings not only advance our understanding of root development but will be valuable in efforts to breed varieties with improved root systems.

## Highlight

MADS-box TF CmANR1 modulates both AR and LR development by directly regulating auxin transport gene *CmPIN2* in chrysanthemum.

## Introduction

Plant roots are crucial to their anchorage, absorption of nutrients and water, as well as to establishment of beneficial symbioses with the surrounding soil microorganism communities^1,2^. In some cases, such as in the dicot model plant *Arabidopsis*, the radicle is generated during embryogenesis within a seed. Following germination, the radicle elongates as the primary root (PR), and typically grows into a central taproot together with the sequential development of the associated lateral roots (LRs)^3^. However, monocot plants such as rice (*Oryza sativa*) and maize (*Zea mays*) develop a more complicated root system^4^. Apart from a specific embryonic PR and several seminal roots (SRs) in maize, most cereals possess an expanding shoot-borne root system^5^. The postembryonic shoot-born roots, called brace roots (BRs) and crown roots (CRs), are also able to branch developing lateral roots^6^. Moreover, some plants species such as chrysanthemum (*Chrysanthemum morifolium*), African violet (*Saintpaulia* spp.), strawberries (*Fragaria* spp.), and garlic (*Allium sativum*), which propagate vegetatively, firstly develop numerous adventitious roots (ARs) from the basal cuttings or stolons in their typical ecological environments^3^. Successively, LRs or higher-order LRs originate from the existing ARs to expand the root system in order to obtain more water and nutrients.

Adventitious roots, which are similar to lateral roots, develop post-embryonically. ARs usually arise from vegetative organs, such as the stolons, rhizomes, leaves, and stems, while LRs often originate from existing roots, such as the PRs, previous LRs, or ARs^3^. Despite the differences in origin, the formation and development of ARs and LRs is controlled by a suite of similar endogenous and environmental factors^3,7,8^. Among these common regulatory factors, auxin is the most vital regulator of both AR and LR development^9–13^. Natural auxins (e.g., indole-3-acetic acid (IAA)) and synthetic analogs (e.g., indole-3-butyric acid (IBA)) have a powerful and stimulatory effect on rooting in many plant species^12,14,15^. A diverse range of studies have shown that auxin is central to AR and LR development in plants, where it cross-talks with other signals (e.g., calcium signal)^16,17^, regulatory genes (e.g., *AtMYB93*, *SHR*, and *ERF3*)^18–20^, or phytohormones (e.g., ethylene and gibberellins)^21–23^. Furthermore, auxin-related biological processes, such as signal transduction, polar transport, and local biosynthesis, account for the primary underlying molecular mechanisms regulating AR or LR formation^10,11,24–27^.

Generally, auxin is produced in the aerial tissues (such as apical meristems) and is then distributed locally and systemically throughout whole plant via two distinct yet interconnected ways: a direct and fast flow from shoots to roots via the vascular central cylinder, and cell-to-cell active polar transport through the outer layers of the root cells^28,29^. However, auxin is also synthesized in the root tips, where auxin transport is characterized by dual polarities. In the roots, auxin polar transport has been described as acropetally (towards root apex) and basipetally (from apex to base)^30^. The auxin efflux carriers PIN-FORMED (PIN) proteins as well as auxin influx carriers AUXIN RESISTANT1/LIKE AUX1 (AUX1/LAX) proteins have been identified as the main components responsible for auxin transport^31,32^. The asymmetric subcellular distribution and localization of carrier proteins contributes to their polarity^33^, among which PIN polarity has been shown to be the primary direction-determining factor in auxin polar transport^34^. So far, PIN members have mostly been well studied in *Arabidopsis* where they exhibit unique but somewhat overlapped localization in various cell types. PIN1 localizes in the basal end of the vascular cells, facilitating the root-ward movement of auxin^35^. PIN2 predominantly resides basally in cortical cells, and apically in the epidermal and root cap cells^34,36^. PIN3 has been detected in the columella cells of the roots in an apolar manner. PIN7 localizes basally in the stele cells in the meristem and elongation zone^37^. Recent studies reported that PIN5, PIN6, and PIN8 located on both the endoplasmic reticulum and plasma membrane^38–42^. Additionally, *PINs* have been functionally identified as vital regulators of numerous auxin-related developmental processes. For instance, *OsPIN1* takes part in auxin-dependent AR emergence and development in rice^43^. *OsPIN2* regulates tiller angle, number, as well as plant height by enhancing basipetal auxin transport in rice^44^. In *Arabidopsis*, the triple mutant *pin1 pin3 pin4* is defective in PR development^33^; *PIN6* is required for LR and AR organogenesis by controlling auxin homeostasis and distribution^42^; and PIN8 exerts a crucial role on pollen development and functionality^38,39^.

Transcription factors (TFs) represent a large portion of the essential regulators of many developmental aspects in plants. With respect to root development regulation, a multitude of TFs have been reported to participate in, and influence, a diverse set of developmental stages of different root types in many plant species^18–20,45,46^. The MADS-box TFs became a point of interest into the genetic regulation of root development. In addition to developmental regulation of flower, fruit, seed, and leaf developmental regulation in plants^47–50^, an increasing number of MADS-box genes have been reported to be involved in root system development. For example, *AtANR1* was the first MADS-box TF gene identified to regulate LR elongation under heterogeneous nitrate conditions^51^. The MADS-box gene *XAL1/AGL12* gene was determined to be necessary for normal root development and growth via a positive control on cell cycle components^52^. *XAL2/AGL14* plays an essential role in robust root patterning by modulating auxin polar transport^53^. *AGL21* was found to be a positive regulator of LR initiation and growth by increasing local auxin biosynthesis in *Arabidopsis*^27^. The *ANR1*-like gene *OsMADS25* positively regulates both PR and LR development by promoting nitrate accumulation in rice^54^. *GmNMHC5*, a MADS-box TF gene in soybean, promotes LR development in a sucrose-dependent manner^55^.

Recently, it was reported that the ectopic expression of *CmANR1*/*CmAGL44* in *Arabidopsis* could promote LR development^56^. However, the underlying mechanisms of *CmANR1* modulation of LR development are largely unknown. Here, it was found that CmANR1 positively modulates AR and LR development by directly regulating *CmPIN2* in chrysanthemum. The potential application of the *CmANR1* gene in controlling root system development and its theoretical research value in breeding programs in chrysanthemum are discussed in this paper.

## Materials and methods

### Chrysanthemum and growth conditions

The *35S::CmANR1*-1258 (green fluorescent protein (GFP) tag) overexpressed vector was constructed as previously described^56^. It was then transformed into *Agrobacterium* strain GV3101. The wild-type (WT) tissue-cultured chrysanthemum were kindly provided by Professor Gao (China Agricultural University). The *CmANR1*-transgenic chrysanthemum were obtained by *Agrobacterium*-mediated transformation of leaf discs^57^. In tissue-cultured condition, *CmANR1*-transgenic and WT plants were cultivated in vitro on Murashige and Skoog (MS) medium in the standardized culture room.

In hydroponic-cultured condition, the chrysanthemum were cultivated in improved Hogland nutrient solution (CaCl_2_ 555 mg/L, MgSO_4_•7H_2_O 493 mg/L, KH_2_PO_4_ 136 mg/L, FeSO4•7H_2_O 27.6 mg/L, EDTA-2Na 3.73 mg/L, KNO_3_ 10 mM, H_3_BO_4_ 2.86 mg/L, MnCl_2_•4H_2_O 1.82 mg/L, ZnSO_4_•7H_2_O 0.23 mg/L, H_2_MoO_4_•H_2_O 0.09 mg/L, CuSO_4_•5H_2_O 0.08 mg/L, pH = 5.6). A simple aeration device was used to supply oxygen in hydroponic condition in case that roots would go rotted.

### *Arabidopsis* AR rooting assays

The *Arabidopsis* lines used here were the *CmANR1*-overexpressing (OE) lines and the “*Columbia*” ecotype. For the procedure of seed sterilization and the growth conditions of *Arabidopsis*, refer to our previous study^56^. After sterilization, the seeds were planted on MS medium with 1% (w/v) sucrose and 0.7% (w/v) agar. Then, the seedlings with only two cotyledons on were trimmed, leaving only the hypocotyls. The hypocotyls were transferred vertically on either MS or MS added with 0.1 μM IBA for AR rooting analysis. The AR rooting assays were performed in a dark growth incubator (23 ± 1 °C, 40% relative humidity) for a week.

### Morphological characterization of roots in chrysanthemum and *Arabidopsis*

The 20-day-old in vitro chrysanthemum and 40-day-old hydroponic-cultured chrysanthemum were used for root morphological characterization. The relevant root data such as root total length, root volume, and root surface were analyzed by WinRHIZO software (Regent Instruments Inc., Canada). The root numbers of AR and LR were counted using Image J software (NIH, Bethesda, MD, USA) of digital images of roots. The root morphology of *CmANR1*-overexpressing and WT control *Arabidopsis* seedlings was on observed MS solid medium with 0.8% agar. Photos of the seedlings were taken after 1 week of darkness, about 10 days old. AR number and AR length was measured by hand using Image J software (NIH, Bethesda, MD, USA).

### RNA-Seq data processing, de novo assembly, and annotation

The samples were the 40-day-old hydroponic-cultured *CmANR1*-overexpressing plants (*CmANR1*-*OVX56*, -*OVX67*, -*OVX81*) and WT plants. Three independent plants of each line consisted of the triplicate samples. Total RNAs of the samples were extracted using the RNeasy plant mini kit (New England Biolabs Inc., New England). The complementary DNA (cDNA) library was prepared as described by Grabherr et al.^58^ and the sequencing was performed on the Illumina HiSeq Platform (Ori-gene Inc., Beijing, China).

Raw RNA-Sequencing (RNA-Seq) reads were conducted with Cutadapt based on BMA algorithm to remove sequence artifacts such as adapter sequences on both ends, low-quality trailing (Q_30_), 3’-end barcode sequences, and reads with lengths less than 60 bp. The remaining valid cleaned reads were processed into de novo transcript assembly according to a previous study^58^. Furthermore, the resulting reads were assembled using iAssembler with a threshold of (-*p*) set to 99^59,60^.

The resulting unigenes were screened by BLAST (Basic Local Alignment Search Tool) against the GenBank non-redundant (NR), TrEMBL, Swiss-Prot, Pfam, and KOG. The unigenes with a cutoff of *E*-value of ≤1e−5 and ≥30% identify were needed for further functional annotation. The chrysanthemum assembled unigenes and their corresponding homologs in the UniProt database were assigned to Gene Ontology (GO) terms. Biochemical pathway prediction of the chrysanthemum transcripts were annotated and enriched by the Pathway Tools^61,62^.

### Quantitative real-time (qRT)-PCR analysis

Total RNA was extracted from the roots of 40-day-old hydroponic-cultured transgenic and WT chrysanthemum using the RNA plant plus Reagent (Tiangen, Beijing, China).Then, cDNA was synthesized using the PrimeScript first-strand cDNA synthesis kit (TaKaRa, Dalian, China). Each qRT-PCR reaction (20 μl) included 1 μl cDNA template, 1 μl of both up and down primers (10 μM), 10 μl SYBR Green Ι, and 7 μl RNase-free H_2_O. The qRT-PCR assays were carried out according to the StepOne real-time PCR system (Applied Biosystems). All reactions were repeated three times, and a chrysanthemum *Ubiquitin* gene (*CmUBI*) served as the reference gene^60^. Relative gene transcript abundances were computed with the 2^−ΔΔCt^ method^56^. The primers used for qPCR reactions are referred to in Supplementary Table 4.

### The cloning and analysis of the promoters

Genomic DNA extracted from the leaves of “*Jinba*“ using the Plant Whole-genome Extraction Kit (Tiangen, Beijing, China) served as the PCR template. The promoters of four auxin-responsive genes *pCmPIN2*, *pCmGH3.1*, *pCmTAA1*, and *pCmAB37G* were cloned according to the instructions of Genome-walking Kit (TaKaRa, Dalian, China). A length of 1994 bp *pCmPIN2* was a twice genome-walking result. The specific primers for cloning the promoters were designed according to cDNA sequences searched in our RNA-Seq result. Related primers are listed in Supplementary Table 4. PLACE *cis*-acting regulatory DNA elements analysis was completed on the following website (http://bioinformatics.psb.ugent.be/webtools/plantcare/html/).

### The expression and purification of CmANR1-His fusion protein

The open reading frame (ORF) of *CmANR1* was cloned from the cDNA of *CmANR1* using the paired primers with *Bam*HI and *Xho*I sites (Supplementary Table 4), and then was constructed into the pET-32a vector, which had a histone (His) tag sequence. Then, the recombinant plasmid was introduced into *Escherichia coli* BL21 (DE3). The CmANR1 (His)-BL21 bacteria was incubated at 37 °C constant temperature shaker (200 rpm) for about 2 h. Subsequently the bacteria were treated with 3 mM isopropyl β-d-1-thiogalactopyranoside (IPTG) for inducing the generation of the CmANR1-His fusion protein. After 4 °C centrifugation of the bacteria, the precipitate was denatured and renatured with a series of specific concentrations of urea solution^63^. The final fusion protein was transferred to a cobalt chelate affinity resin, which contained the immobilized His-tag. The tube was incubated at 4 °C for 2 h on the shaker. After three times separation and abstersion, the protein was collected and detected by western blot using His antibodies (Abcam, Cambridge, UK).

### ChIP-qPCR and EMSA analysis

The chromatin immunoprecipitation quantitative PCR (ChIP-qPCR) tests were performed using the EpiTect ChIP OneDay kit (QIAGEN,Shanghai, China) as described in the previous study^63^. The primers used for ChIP-qPCR are described in Supplementary Table 4. Gel electrophoresis mobility shift assay (EMSA) was carried out following the instructions of the manufacturer in the Light Shift Chemiluminescent EMSA Kit (Thermo, Waltham, MA, USA). Concisely, the biotin-labeled probe was incubated in the 1× gel/DNA shift binding buffer containing 5 mM MgCl_2_, 50 mM KCl, 2.5% glycerol, and 10 mM EDTA with or without CmANR1 protein at 24 °C for 25–30 min. The unlabeled probe with specified concentrations (50×, 100×) was used for cold probe competition. Related primers are referred to in Supplementary Table 4.

### In vivo firefly luciferase (Luc) imaging assay

The Luc imaging assays were carried out in *Nicotiana benthamiana* leaves, while the transient expression was performed as previously described^64^. The promoter of *CmPIN2* was cloned into pGreenII 0800-LUC vector, generating the reporter *CmPIN2*_*pro*_*::LUC*. The effector (*35S*_*pro*_*::CmANR1*) was constructed by cloning the fragment of *CmANR1* (ORF) into the pGreenII 62-SK vector. Then, the recombinant vectors *CmPIN2*_*pro*_*::LUC* and *35S*_*pro*_*::CmANR1* as well as the empty vectors pGreenII 0800-LUC (LUC) and pGreenII 62-SK (35S) were introduced into *Agrobacterium* strain LBA4404, respectively. The four independent *Agrobacterium* bacteria with similar OD_600_ absorbance were 1:1 pairwise mixed. The four kinds of mixed bacteria were infiltrated on the four sites of a same mature *N. benthamiana* leaf, respectively. A fluorescence imaging instrument (NightOWL II LB983) in conjunction with the Indigo software was used for LUC imaging and luminescence intensity quantification. Infiltrated leaves were sprayed with little luciferin (100 mM), then were put in darkness for 5–10 min before LUC imaging^65^. Related primers are listed in Supplementary Table 4.

### Determination of total IAA in roots

About 0.2 g (fresh weight) root samples were prepared and vacuum-dried at −35 °C for about 12 h. After quick grind in liquid nitrogen, the powder of the samples was extracted in accordance with the method described by Lin et al.^66^. The total free IAA was detected by the high-performance liquid chromatography.

### Statistical analysis

All samples were analyzed in at least triplicate repeats and represented as the mean ± standard deviation unless specifically labeled. Significance analysis was determined by Student’s *t*-test. *P* ≤ 0.001 meant a extremely significant difference, *p* ≤ 0.01 represented a significant difference, while n.s. meant no significance.

## Results

### *CmANR1* promotes AR development in chrysanthemum under tissue culture conditions

**Fig. 1:**
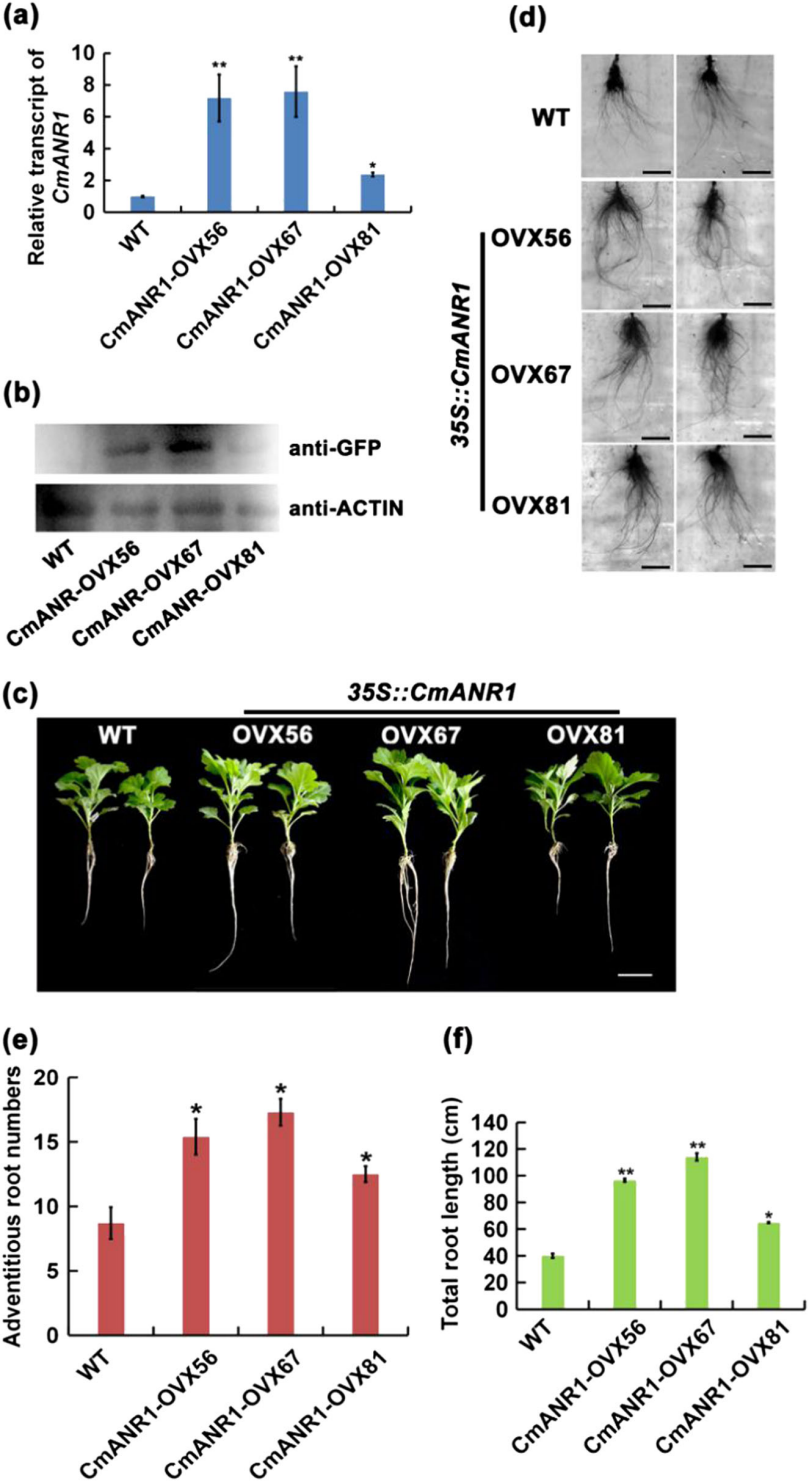
*CmANR1* plays a positive role in adventitious root development in tissue-cultured chrysanthemum. **a** Relative expression level of the *CmANR1* gene in the *CmANR1*-transgenic and the WT chrysanthemum lines. **b** The level of the CmANR1-GFP fusion protein in *CmANR1*-transgenic plants, as determined by immunoblot analysis using an anti-GFP antibody. The anti-actin antibody was used as a loading control. **c** Phenotypes of *CmANR1-OVXs* and WT chrysanthemum (tissue culture). Scale bar = 1 cm. **d** The root-specific scanning pictures of *CmANR1*-transgenic and WT plants (tissue culture). Scale bar = 1 cm. **e** AR number and total root length (**f**) of *CmANR1*-transgenic and WT plants (tissue culture). Data are shown as the mean ± standard error (SE) based on three replicate. Statistical significance was determined using Student’s *t*-test. No significance (n.s.): *p* > 0.01; *p < 0.01; **p < 0.001

The *35S::CmANR1*-1258 (GFP) recombinant plasmid was introduced into chrysanthemum leaf discs using *Agrobacterium GV3101*-mediated transformation. Subsequently, several positive candidates of *CmANR1*-transgenic plants, which were preliminarily screened by PCR detection using the genomic DNA as the template, were further identified by qRT-PCR analysis (Supplementary Fig. S1). Among them, three independent *CmANR1*-transgenic lines (*CmANR1*-*OVX56*, -*OVX67*, -*OVX81*) with significantly distinct expression levels of *CmANR1* were selected for further investigation (Fig. 1a). Immunoblotting assays showed that the CmANR1 protein accumulated in these three *CmANR1*-transgenic chrysanthemums much more than in the WT control (Fig. 1b). These three *CmANR1*-transgenic and WT chrysanthemums were then rooted and cultivated in vitro. Following this, the *CmANR1*-overexpressing plants exhibited a stimulation on AR development compared with the WT plants (Fig. 1c, d). Remarkably, the numbers and total lengths of the ARs in the *CmANR1*-*OVXs* lines were increased by 43.7–98.8% and 61.8–184.5%, respectively, in comparison to the WT plants (Fig. 1e, f). Therefore, a significant increase in both root surface and volume was showed in the transgenic plants compared to the WT plants (Supplementary Table 1). These results suggest a positive role of *CmANR1* on AR development in chrysanthemum.

Surprisingly, fewer LRs were found in both the *CmANR1*-transgenic and WT plants under tissue culture conditions, which seemed to be in contrast to the positive role of *ANR1* on LR growth in *Arabidopsis*^51,56^. To further confirm the role of *CmANR1* on AR development, AR rooting experiments were performed using *CmANR1*-overexpressing (*CmANR1*-OE3, OE6 and OE9) and WT *Arabidopsis* seedlings, which were obtained in our previous study^56^. Only the hypocotyls of those plants were placed on the MS medium and the MS medium with 0.1 µM IBA added, and cultivated vertically under dark conditions. Following this, the seedlings developed new ARs after about 1 week of growth. The total length and numbers of ARs in the *CmANR1*-transgenic seedlings were significantly increased compared to the WT plants, with respective increases of 8.8–56.6% and 50.4–150.2% (Supplementary Fig. S2). Additionally, the exogenous application of IBA almost abolished the developmental differences of the ARs between the transgenic and WT seedlings, suggesting that auxin may have some relationship with AR development.

### *CmANR1* promotes AR and LR development in chrysanthemum under hydroponic culture conditions

**Fig. 2:**
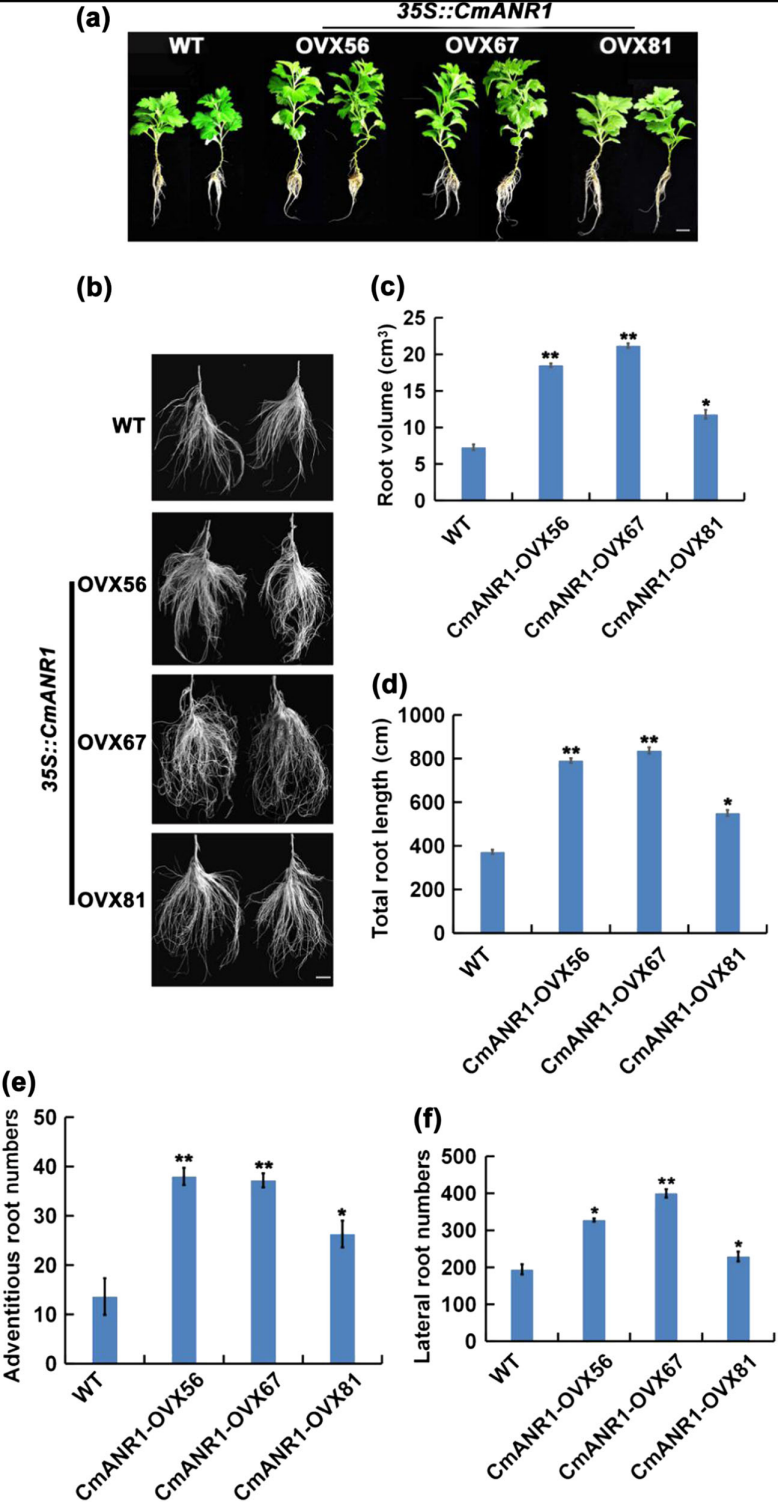
*CmANR1* promotes both adventitious root and lateral root development in hydroponic-cultured chrysanthemum. **a** Phenotypes of *CmANR1*-transgenic and WT chrysanthemums under hydroponic culture conditions. Scale bar = 1 cm. **b** The root-specific magnified pictures of *CmANR1*-*OVXs* and WT plants (hydroponic culture). Scale bar = 1 cm. **c**–**f** The root volume (**c**), total root length (**d**), AR number (**e**), and LR number (**f**) of *CmANR1*-transgenic and WT plants. The data represent the means ± SE of three independent experiments. Statistical significance was determined using Student’s *t*-test. n.s.: *p* > 0.01; *p < 0.01; **p < 0.001

To better evaluate the function of *CmANR1* in root system development, the WT and *CmANR1*-overexpressing chrysanthemums exhibiting uniform growth under tissue-cultured conditions were then cultivated hydroponically. The results showed that the *CmANR1*-overexpressing chrysanthemums possessed a much more extensive root system, including more ARs and LRs, compared to the WT plants after about 35–40 days of growth (Fig. 2a, b). *CmANR1*-overexpressing chrysanthemum exhibited a significant increase in root volume and total root length compared to the WT plants, by 0.6–1.9-fold and 0.5–1.2-fold, respectively (Fig. 2c, d). Meanwhile, the numbers of ARs and LRs in the three *CmANR1*-overexpressing plants were much greater than those of the WT plants, with 0.3–0.5-fold and 0.2–1.1-fold increases, respectively (Fig. 2e, f). The stronger root system of the *CmANR1-*transgenic plants indicated the positive effect of *CmANR1* on rooting in chrysanthemum under hydroponic conditions. Furthermore, a significant stimulation on shoot height was observed in the *CmANR1*-overexpressing chrysanthemums compared to the WT plants, elevated by 16.1–51.9% (Supplementary Table 2). The increase in shoot height of the *CmANR1-OVXs* plants may be attributed to a feedback-enhanced uptake of water and nutrients by the more extensive root system.

### Transcriptome sequencing of *CmANR1*-overexpressing and WT chrysanthemum roots

To reveal the underlying mechanism of *CmANR1* in controlling root system development, strand-specific RNA sequencing libraries from the roots of the WT and *CmANR1*-overexpressing chrysanthemums were constructed. De novo assembly of the valid cleaned reads produced 51,481 unigenes with a mean length of 653 bp and a longest length of 8281 bp. The length distribution of the assembled unigenes is exhibited in Supplementary Table 3. Then, we annotated the assembled unique transcripts by BLAST against several protein databases. A total of 24,292 (47.2%), 36,870 (71.7%), 37,115 (72.2%), 28,442 (55.3%), 30,328 (59.0%), 30,392 (59.1%), and 12,050 (23.4%) unique transcripts obtained significant hits (identity ≥30%, *E*-value ≤ 1e−5) in the Swiss-Prot, TrEMBL, GenBank NR, Pfam, eukaryotic orthologous groups (KOG), GO (http://www.geneontology.org/), and Kyoto Encyclopedia of Genes and Genomes (KEGG) databases, respectively. Remarkably, “Signal transduction mechanisms” was the second-most abundant group in these KOG functional categories after “Posttranslational modification, protein turnover, chaperones”, irrespective of the poorly characterized ones (Supplementary Fig. S3a). In contrast, the GO terms “metabolic process” in the biological process category, “cell” in the cellular category, and “binding” in the molecular function category were the most enriched in these three categories, respectively (Supplementary Fig. S3b). In the KEGG pathway classification, “Carbohydrate metabolism” in the metabolism category was the most abundant group (Supplementary Fig. S3c).

**Fig. 3:**
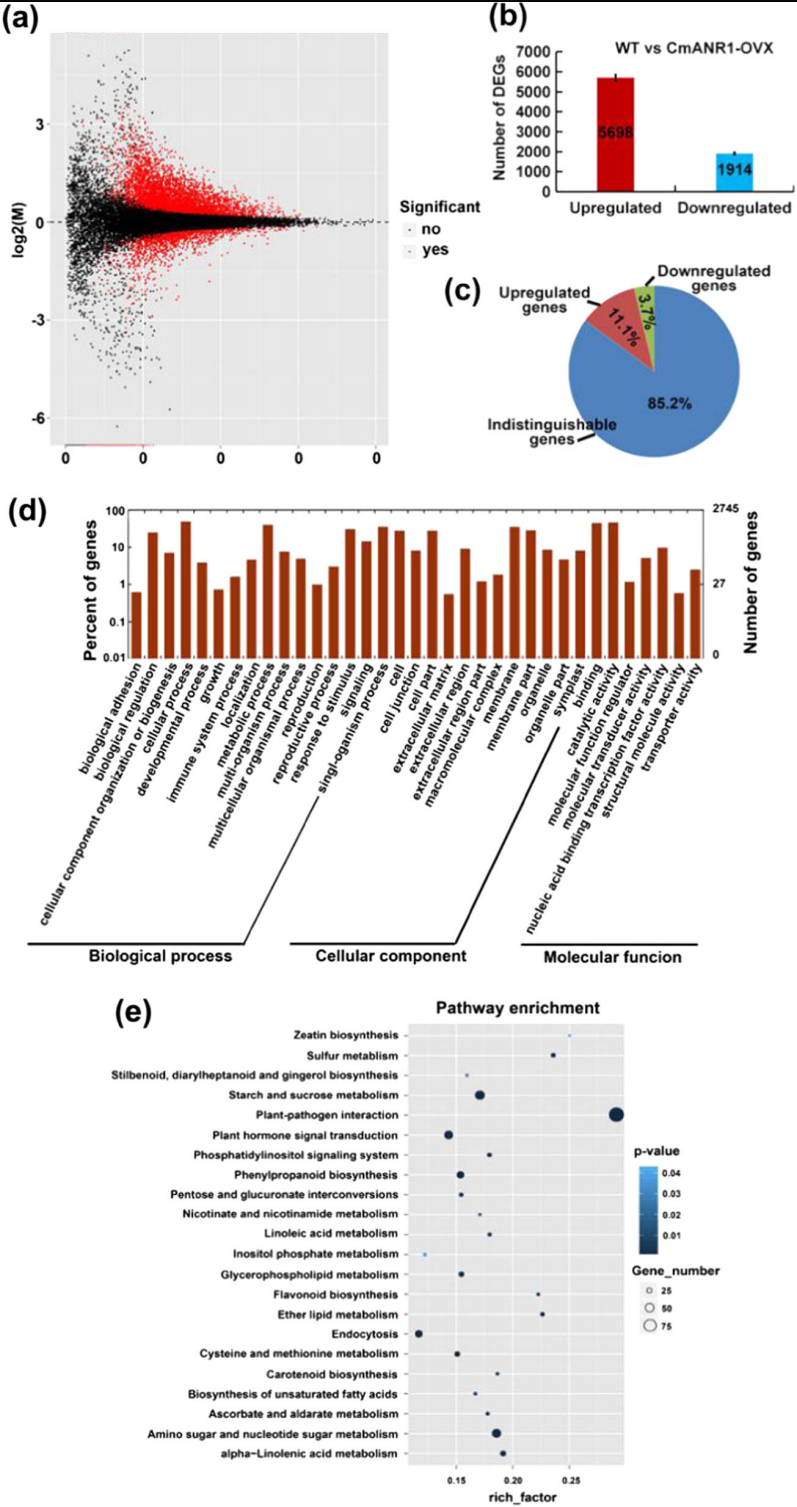
Annotation enrichment of DEGs in the roots of *CmANR1*-*OVXs* in contrast with WT chrysanthemum. **a** The volcano plot of the DEGs. A small dot in the picture represents a unigene; the *x*-axis represents the log_2_(fold change) of a gene expression difference between WT and *CmANR1-OVXs*; the *y*-axis represents the negative log Kow of the *p* value of the gene expression change. Unigenes with significant differential expression are indicated with a red dot. **b** The numbers of up-regulated and down-regulated unigenes in the RNA-Seq results. **c** Pie chart representing the percentage of up- and down-regulated unigenes and indistinguishable unigenes in RNA-Seq data. **d** GO enrichment analysis of the DEGs. The *x*-coordinate is the GO classification, which is the secondary function of GO; the left vertical coordinate is the percentage of the DEGs; and the right shows the corresponding numbers. **e** KEGG bubble chart of the DEGs. The bubble size represents the number of DEGs, and the bubble color represents the *p* value. The rich-factor equals the number of DEGs/the number of background genes in a certain signaling pathway

Subsequently, FPKM (Fragments Per Kilobase of transcript per Million fragments mapped) was used to evaluate the expressional abundances of the assembled unigenes based on the transcriptome sequencing data. The volcano plot showed the relationship between the significance of the *p* value and fold change of all the differentially expressed unigenes (DEGs) (Fig. 3a). Simultaneously, the MA Value Plot presented the distribution and differences in DEGs in the WT and *CmANR1*-overexpressing chrysanthemums (Supplementary Fig. S4a). In addition, a heatmap of the DEGs provided a visual illustration of the expressional differences between the WT and *CmANR1*-overexpressing chrysanthemums, with high expression levels shown in red and low expression levels shown in green (Supplementary Fig. S4b). A total of 7612 DEGs were identified in the *CmANR1*-overexpressing chrysanthemums compared to the WT plants (Fig. 3b; Supplementary Appendix S1). Among them, 5698 (11.1%) were up-regulated and 1914 (3.7%) were down-regulated (Fig. 3b, c; Supplementary Appendix S1). Subsequently, these up-regulated and down-regulated genes that were involved in the main biological functions, as well as the biochemical metabolic and signal transduction pathways in GO and KEGG enrichment, were analyzed (Supplementary Appendix S2 and S3). GO terms involving “catalytic activity” and “binding” in the molecular function category and “metabolic process”, “response to stimulus”, and “signaling” in the biological process category were highly abundant in the GO enrichment annotation of the up-regulated unigenes (Fig. 3d). Meanwhile, “Plant hormone signal transduction” was abundant in the KEGG-enrich analysis and enriched bubble diagram of the up-regulated unigenes (Fig. 3e). These information suggested that *CmANR1* might participate in the regulation of plant hormone signaling processes. However, DEGs being classified to “Plant-pathogen interaction” term in KEGG-enrich analysis accounted for the most abundant, which attracted our attention and would be discussed later.

### Expressional profiling of the genes involved in root system development in chrysanthemum

**Fig. 4:**
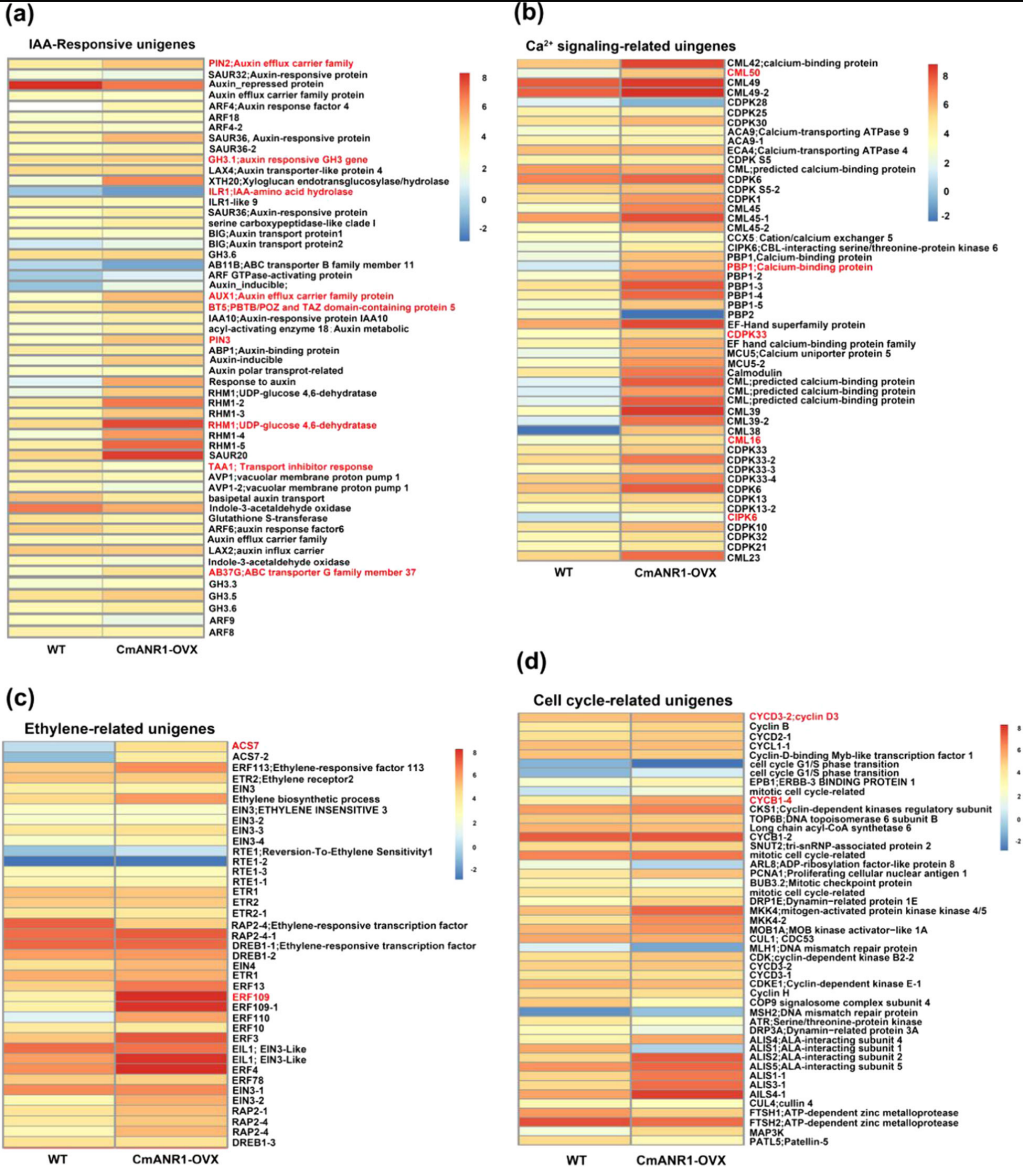
Heatmaps of four groups of candidate unigenes associated with root system development in the DEGs. **a** Heatmap of auxin-responsive unigenes. **b** Heatmap of Ca^2+^ signaling-related unigenes. **c** Heatmap of ethylene-related unigenes. **d** Heatmap of cell cycle-related unigenes. Differences in expression levels are represented by color gradients. Red and orange strips indicate highly to moderately up-regulated unigenes, while dark blue to light blue strips represent highly to moderately down-regulated unigenes. The ID numbers of the four groups of unigenes are provided in Supplementary Appendix S4. The red unigenes in the four groups were selected for qRT-PCR

Four groups of potential candidate unigenes from the DEGs associated with root system development, including auxin-responsive group, calcium (Ca^2+^) signaling-related group, ethylene-related group, and cell cycle group, were selected for further investigation. The corresponding ID numbers and log_2_(fold changes) values of these unigenes are listed in a supplementary file (Supplementary Appendix S4). The heatmaps display the average absolute expression values after log_2_ transformation between WT and *CmANR1*-overexpressing chrysanthemums (Fig. 4). Among them, the expressions of some auxin transport unigenes (*PIN2*, *AUX1*, *AB37G*, and *AB11B*) and a range of auxin-responsive protein genes (*SAU20*, *SAU36*, *RHM1*, and *XTH20*) showed a significant increase in the *CmANR1*-overexpressing chrysanthemum compared to the WT plants in the auxin-responsive group (Fig. 4a). Ca^2+^ signaling-related unigenes, such as the calcium-binding protein genes *CML*s (*CML45*, *CML23*, and *CML50*) and calcium-dependent protein kinase genes *CDPKs* (*CDPK6*, *CDPK10*, *CDPK30*, and *CDPK33*), were also found to be significantly up-regulated in the *CmANR1*-overexpressing chrysanthemums compared to the WT plants (Fig. 4b). The ethylene biosynthesis genes *ACS7* and ethylene-responsive TF genes *ERFs* (*ERF3*, *ERF78*, and *ERF109*) were significantly up-regulated in the *CmANR1*-overexpressing chrysanthemums compared with the WT plants (Fig. 4c). In addition, the expressions of cell cycle-related genes (*CYCB1-4*, *CYCD3-2*, *ALISs*, *MLH1*, and *MKK4*) were also significantly altered in the *CmANR1*-overexpressing chrysanthemums compared to the WT plants (Fig. 4d).

**Fig. 5:**
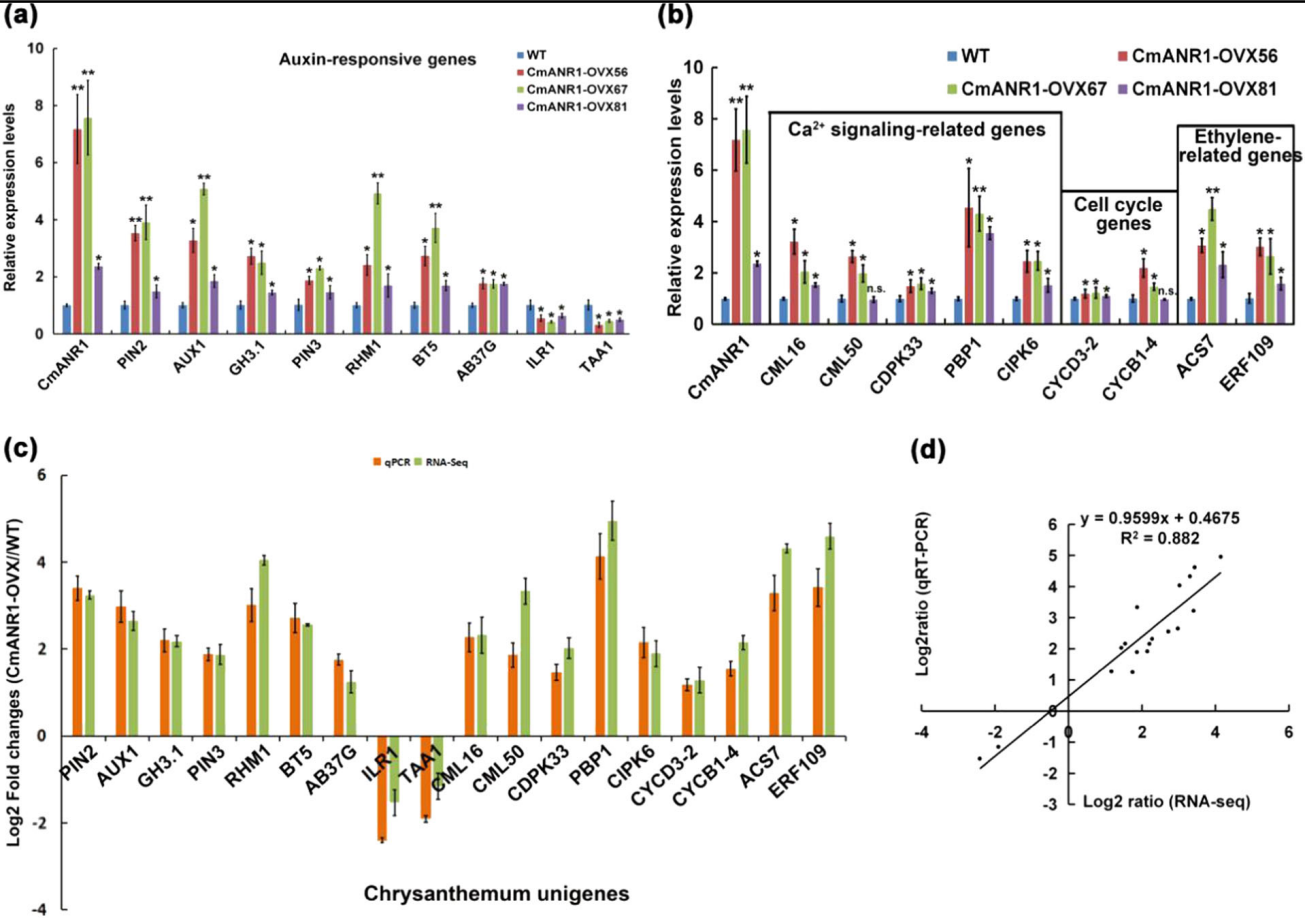
Verification of the RNA-Seq results by qRT-PCR. **a** The relative transcript abundance of some IAA-responsive unigenes that were selected based on their differential expression in the roots of WT *vs. CmANR1*-transgenetic plants. **b** The relative expression level of the unigenes in Ca^2+^ signaling-related, ethylene-related, and cell cycle-related groups in the roots of WT and *CmANR1*-transgenetic plants. **c** Comparison of the expression level of unigenes between the RNA-Seq and qRT-PCR. **d** Scatter diagram of the log ratios (log_2_ FC) of the unigenes. The qRT-PCR data were normalized to the internal control *CmUBI*. Note that in **a** and **b**, data are shown as the mean ± SE based on three or more replicate. Statistical significance was determined using Student’s *t*-test. n.s.: *p* > 0.01; *p < 0.01; **p < 0.001

To further validate our RNA-Seq results, 18 unigenes that were marked in red in Fig. 4 from the four groups were selected for qRT-PCR verification. As a result, most of these unigenes were significantly increased in the *CmANR1*-overexpressing chrysanthemums compared to the WT plants (Fig. 5a, b). Additionally, we found that the exact fold changes of the detected unigenes varied between the qPCR analysis and RNA-Seq expression data (Fig. 5c), but the high correlation (*R*^2^ = 0.882) correlated with a simple linear regression equation *y* = 0.96*x* + 0.467 demonstrated that a good consistency existed between the two experimental methods (Fig. 5d).

### CmANR1 facilitates auxin polar transport by direct transcriptional activation of *CmPIN2*

**Fig. 6:**
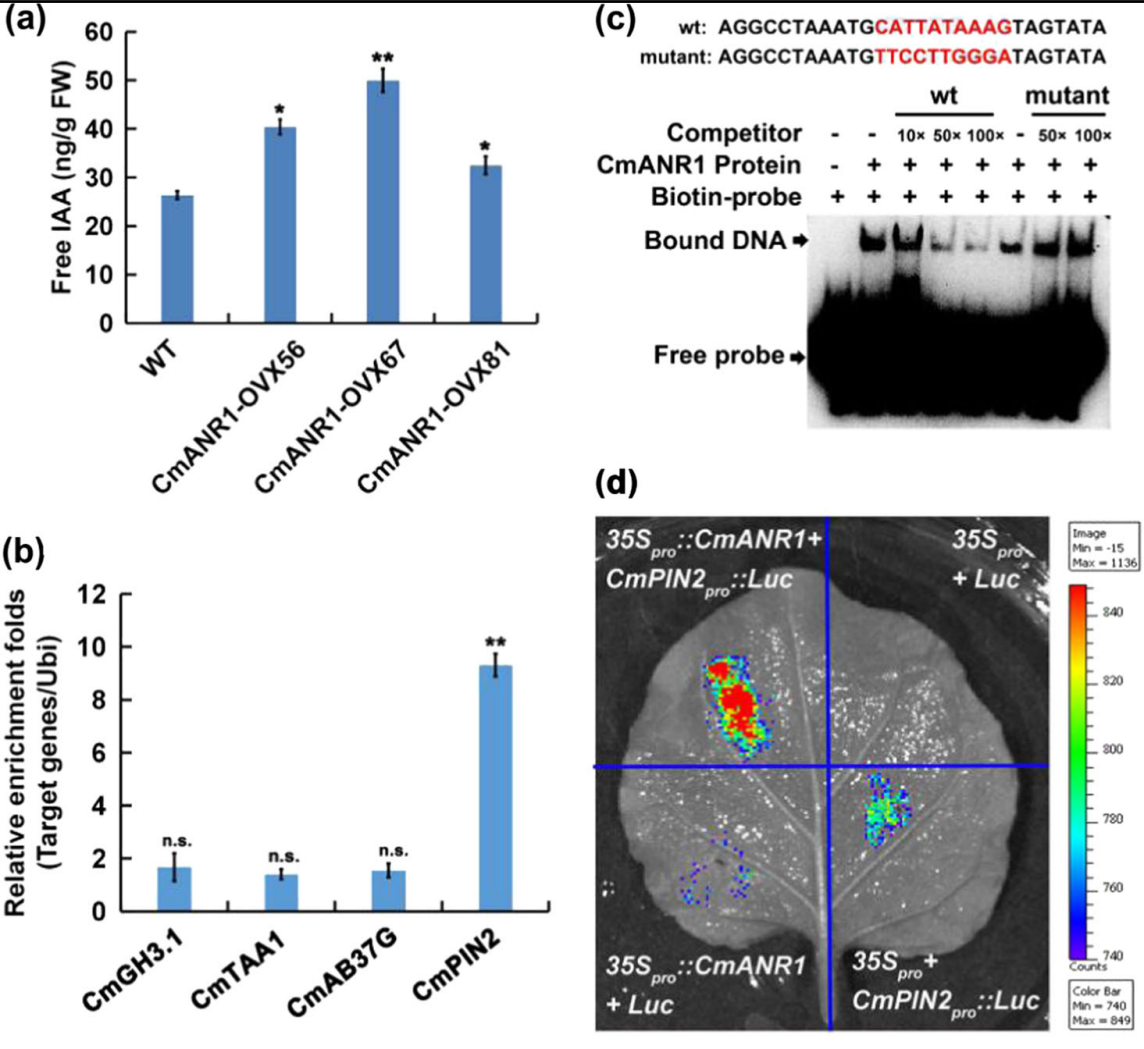
CmANR1 binds directly to the *CmPIN2* promoter. **a** Total free IAA content in the roots of 20-day-old WT and *CmANR1*-transgenic chrysanthemums. **b** The relative enrichment folds of the fragment containing the CArG-box motif by ChIP-qPCR in the promoters of four auxin-responsive unigenes. **c** CmANR1 directly binds to the CArG motif presented in *pCmPIN2* in vitro, as indicated by EMSA. Competition for CmANR1 binding was performed with 10×, 50×, and 100× unlabeled probes (wt) or 50× and 100× CArG-box-mutated probes (mut). The symbol “+” indicates presence and “−” indicates absence. **d** The Luc imaging assay indicates that CmANR1 activates the expression of *CmPIN2*. Representative images of *Nicotiana benthamiana* leaves 72 h after infiltration are shown. Statistical significance was determined using Student’s *t*-test. n.s.: *p* > 0.01; *p < 0.01; **p < 0.001

Accumulating evidence suggests that auxin plays a central role in both AR and LR development^30^. To determine the causality between auxin and root system development, we measured the endogenous free IAA content in the roots of the WT and *CmANR1*-transgenic plants. As observed with the up-regulated expressions of auxin polar genes, such as *PIN2*, *PIN3*, and *AUX1* (Fig. 5a), the free IAA level in the roots of *CmANR1*-transgenic chrysanthemum was elevated by 23.4–89.4% compared to the WT plants (Fig. 6a), suggesting that auxin was the underlying cause of the more robust root system in the *CmANR1-*transgenic plants.

MADS-box TFs bind to specific DNA sequences with an overall consensus of CC(A/T)_6_GG, called CArG-box motifs^67^. To further verify whether CmANR1 directly activates the expression of auxin-responsive genes, the promoters of *CmPIN2*, *CmGH3.1*, *CmTAA1*, and *CmAB37G* were cloned by the Genome-Walking method. Plant *cis*-acting regulatory DNA elements (PLACE) analysis found that there was one CArG-box motif in the promoters (Supplementary Fig. S5). Subsequently, the ChIP-qPCR assays were carried out using *CmANR1-*transgenic and WT chrysanthemum. The results demonstrated that the CArG-box motif of *CmPIN2* was significantly recruited by CmANR1, while others were not (Fig. 6b). The results provided in vivo evidence for the binding of CmANR1 to the *CmPIN2* promoter on the CArG-box motif site.

To further validate the binding of CmANR1 to the CArG-box recognition site in the *CmPIN2* promoter in vitro, EMSA analysis was conducted with an oligo-probe containing the CArG-box motif using the purified CmANR1-His fusion protein. The result showed that specific DNA-CmANR1 protein complexes were detected when the CArG-box motif-containing sequence was used as the labeled oligo-probe. When increasing the amounts of the unlabeled competitive probe with the same sequence, we found that the binding complexes were reduced. However, the competition was not existed in the mutated version. The specificity of the competition verified the physical interaction between the CmANR1 protein and *CmPIN2* promoter, which required the specific CArG-box *cis*-element (Fig. 6c).

To examine whether CmANR1 directly activates *CmPIN2*, an in vivo firefly Luc imaging assay was performed. Constructs containing *35S*_*pro*_:*:CmANR1* (pGreenII 62-SK) and the *CmPIN2*_*pro*_*::Luc* (pGreenII 0800-Luc), as well as *Luc* (pGreenII 0800-Luc) and *35S*_*pro*_ (pGreenII 62-SK) were respectively co-infiltrated into tobacco leaves to express these fusion proteins transiently. The results demonstrated that a strong luminescence signal was detected in the *35S*_*pro*_*::CmANR1*/*CmPIN2*_*pro*_*::Luc* coexpression region, but no or very weak luminescence signal was detected in the negative controls (Fig. 6d). These results indicate that CmANR1 directly activates *CmPIN2* transcription.

Taken together, our results suggest that CmANR1 activates the transcription of the *CmPIN2* gene by direct binding to the CArG-box motif in its promoter.

## Discussion

MADS-box TF genes have been extensively identified as the important regulators of flowering time, floral organ identity, and flower development^68–72^. In contrast, fewer MADS-box TF genes have been reported to regulate root development^27,55,56^. With regards to the molecular regulatory mechanisms on root development, MADS-box TF genes are seldom an immediate regulator. In fact, these MADS-box genes indispensably exert their effects on root development through cross-talks with other signals, such as the cross-talk between *OsMADS25* and the nitrate signal^54^, and *GmNMHC5* with the sucrose signal in controlling root development^55^. Remarkably, the functional mechanism of a large part of MADS-box genes on root development is mainly auxin dependent, such as *ANR1*, *AGL21*, and *XAL2/AGL14*^27,52,53^. Interestingly, our previous study on *CmANR1* demonstrated that a nitrate signaling pathway as well as auxin-related processes interacted under the integration of the *CmANR1* gene, giving rise to the proliferation of LR growth in *Arabidopsis*^56^.

A great deal of evidence suggests that auxin is central to both LR development^9,13^ and AR formation^11,12^. In this study, the free IAA content was highly elevated in the roots of *CmANR1-OVXs* plants compared to the WT control (Fig. 6a), which provided a reasonable explanation for the better developed ARs and LRs in *CmANR1*-transgenic chrysanthemums. It is well known that *PINs* and *AUX/LAXs* are two dominating groups of auxin efflux/influx carrier genes that are essential for auxin polar transport in plants^32^. Moreover, *PIN* genes have been reported to provide rate-limiting functions in auxin movement^29,34^. In our study, the expressions of the auxin transport genes *PIN2*, *PIN3*, and *AUX1* were significantly up-regulated in the roots of the *CmANR1-*transgenic plants compared to the WT control (Fig. 5a). Although auxin is also synthesized in the root tips, the several included auxin synthesis unigenes (e.g., *YUC1*, *AAO-Like*, *AAO2*, *NIT2-Like*, and *NIT4*) in the RNA-Seq results showed no obvious expressional differences in the roots between WT and *CmANR1-OVXs*. Given the role of polarity in auxin transport, the increased auxin in the roots of the *CmANR1*-transgenic plants was more likely due to shoot-to-root auxin transport under the mediation of auxin transport carrier genes, but not as a direct result of auxin biosynthesis in the roots. Furthermore, CmANR1 could directly activate *CmPIN2* transcription (Fig. 6), confirming the possibility of root-ward auxin transport at least partially by *CmPIN2* in *CmANR1*-transgenic chrysanthemum.

In addition, calcium, as an important signaling messenger, was found to be related with the regulation of AR or LR development via cross-talks with auxin. Ca^2+^ and CDPK act as the downstream messengers during auxin-induced AR formation in cucumber^16^. *AtCIPK6* significantly affects LR formation by positively regulating root basipetal and shoot-to-root auxin transport^73^. *OsCBL1* is required for LR development in rice by mediation of auxin biosynthesis^17^. In line with previous studies, a multitude of Ca^2+^-signaling-related genes, including *CML6*, *CML45*, *CML50*, *CIPK6*, *CDPK10*, and *CDPK33*, as well as *PBP1*, were significantly up-regulated in the roots of *CmANR1*-overexpressing chrysanthemum compared to WT plants (Figs. 4b, 5b), which indicated possible cross-talk of calcium signaling with auxin-related physiological processes in regulation of root development under the mediation of *CmANR1*. Moreover, the complex interaction of auxin with ethylene in root development has been well documented^21,22,74^. Auxin can stimulate ethylene biosynthesis by increasing *ACS* transcription^75^ and positively regulating the ethylene-mediated inhibition of root growth^76^. Ethylene has a positive role in the regulation of auxin synthesis and promotes basipetal auxin polar transport in the roots, resulting in increased auxin as well as a subsequent triggering of ethylene-mediated root growth inhibition^77–79^. Notably, in a present study, the expression levels of numerous ethylene-related unigenes were markedly varied in the roots of *CmANR1*-transgenic plants compared with the WT plants (Fig. 4c), such as *ACS7*, *ERF13*, and *ERF109*. However, the major components in the ethylene signaling pathway, including *ETR1*, *ETR2*, *EIN2*, *EIN3*, and *EIL1*, showed no obvious expressional differences between the WT and *CmANR1-OVX*, suggesting the absence of the ethylene signaling pathway in this context. There seems to be a paradox between the ethylene signaling and RNA-Seq results here. A possible explanation is that the *ERFs* (*ERF13* and *ERF109*) have other functions, or otherwise are not the main determinants involved in the ethylene signaling pathway. In fact, *ERF109*, which is highly jasmonic acid (JA)-responsive, plays an important role in mediating the connection of JA signaling with auxin biosynthesis during LR formation in *Arabidopsis*^80^. Therefore, ethylene biosynthesis rather than signaling may take part in regulating root development in chrysanthemum. Further researches are needed to prove this hypothesis.

In our transcriptome sequencing results, thousands of unigenes were significantly differentially expressed in the root samples of *CmANR1-OVXs* compared to the WT control (Fig. 3). The annotations on these DEGs were highly informative. In addition to the anticipated DEGs, there were some unexpected results that required further assessment. For example, the “Plant-pathogen interaction” term accounted for the most abundant group in the KEGG enrichment analysis on the up-regulated DEGs (Fig. 3e). In terms of pathogen defense in plants, the phytohormone JA naturally becomes a primary consideration. As a biotic and abiotic stress-related hormone, JA is essential for immunity and development in plants. In addition, methyl JA was proved to repress root growth in *Arabidopsis*^81^. Moreover, a previous study on AR formation in *Arabidopsis* reported that the auxin-inducible *Gretchen Hagen3* (*GH3*) genes, *GH3.3*, *GH3.5*, and *GH3.6*, could lower JA content in the roots by down-regulating JA biosynthesis and enhancing JA conjugation. *GH3* genes were found to fine-tune AR formation by a combination of auxin and JA regulatory pathways^11^. Inspired by the above studies, we discovered that the auxin-responsive *GH3* unigenes (*GH3.1*, *GH3.5*, and *GH3.6*) were significantly up-regulated in the roots of *CmANR1-OVXs* plants compared to the WT control. Conversely, three JA-amido synthetase (JAR1) unigenes were down-regulated in our RNA-Seq results. Therefore, based on this observation, JA signaling may possibly be involved in AR development by cross-talks with auxin under the integration of *CmANR1* in chrysanthemum.

**Fig. 7:**
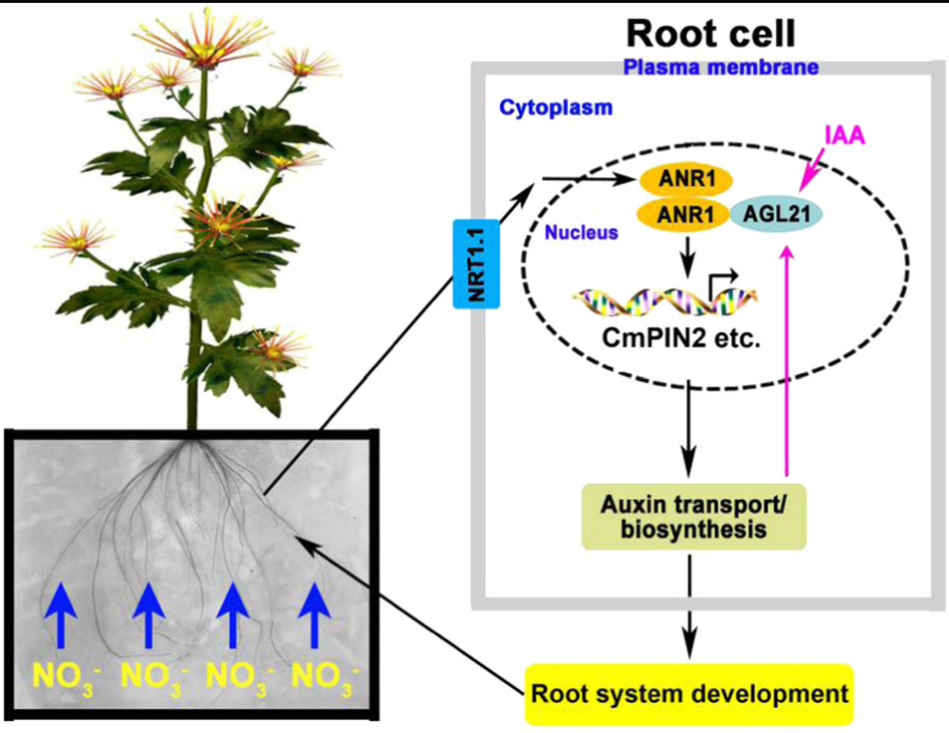
**The working model of** ***CmANR1*** **in root development in chrysanthemum: CmANR1 promotes both AR and LR development by transcriptional activation of** ***CmPIN2*** **in the roots**

In conclusion, we have provided a summary of a working model of the MADS-box TF gene *CmANR1* on the regulation of both AR and LR development in chrysanthemum (Fig. 7). When there was a higher concentration of nitrate in the surviving environment of chrysanthemum roots, NRT1.1, as the nitrate sensor on the root cell plasma membrane, sensed and transferred the external nitrate signal to *ANR1* in the nucleus^82,83^. Then, the nuclear-localized *ANR1* was rapidly enriched in response to the nitrate signal, forming more ANR1/ANR1 homodimers^56^. Subsequently, the TF gene *ANR1* up-regulated the expressions of several auxin polar transport genes, such as *PINs* and *AUX1*, thereby facilitating root-ward auxin transport. Alternatively, in our previous study, local auxin biosynthesis was elevated concurrently^56^. The resulting increased auxin in the roots promoted both AR and LR development in chrysanthemum. In contrast, the increased auxin in the roots may feedback-regulate the expression of a member of the same clade, *AGL2*1, which was shown to interact with *CmANR1*^56^, and *AGL21* exerted similar effects on LR development as *CmANR1*^27,56^. The formation of ANR1/AGL21 heterodimers may then collectively regulate LR development in chrysanthemum. Finally, a robust root system developed in parallel with thriving shoot development and growth in chrysanthemum (Supplementary Table 2).

## Electronic supplementary material

Supplementary Information

Supplementary Appendix S1

Supplementary Appendix S2

Supplementary Appendix S3

Supplementary Appendix S4
