# Supplementary material for: The MADS transcription factor CmANR1 positively modulates root system development by directly regulating CmPIN2 in chrysanthemum
Source: Hortic Res. 2018 Oct 1;5:52. doi: 10.1038/s41438-018-0061-y (PMC6165851; doi:10.1038/s41438-018-0061-y)
Supplement: Supplementary file 1 — Supplementary Information [file 41438_2018_61_MOESM1_ESM.docx]

**Supplementary Information**


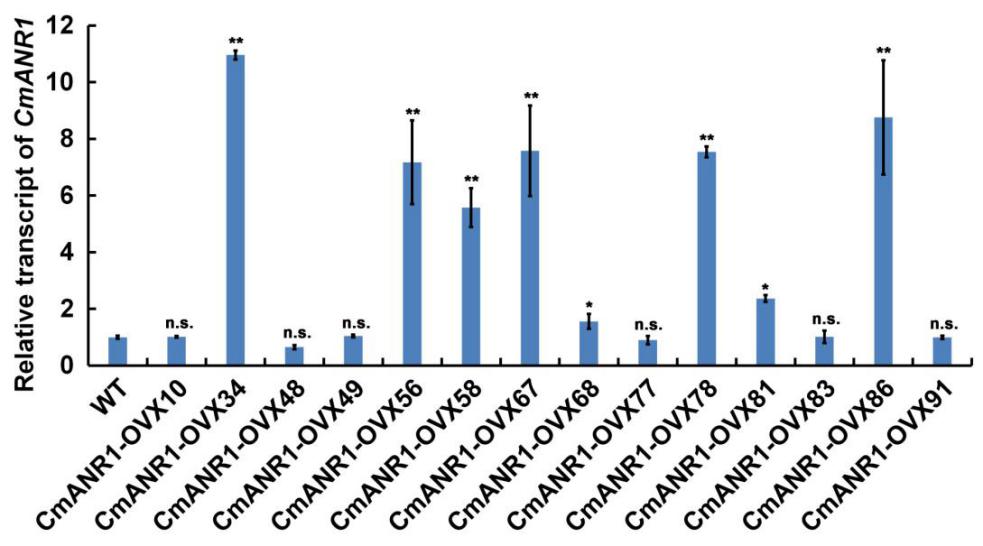


**Supplementary Figure S1.** **The relative transcript level of *CmANR1* in WT and *CmANR1*-transgenic chrysanthemum.** Data are shown as the mean ± SE, based on more than three replicates. Statistical significance was determined using a Student's *t* test. n.s: *P* > 0.01; *: *P* < 0.01; **: *P* < 0.001.


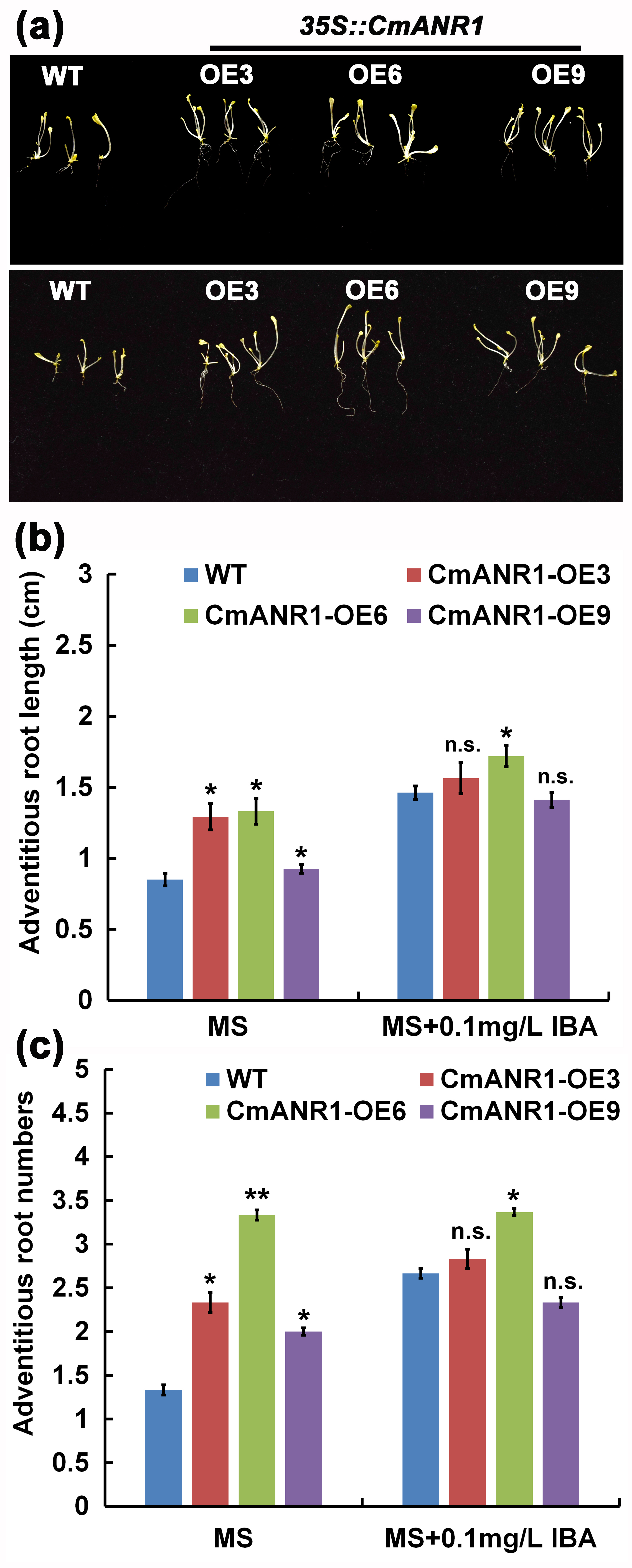


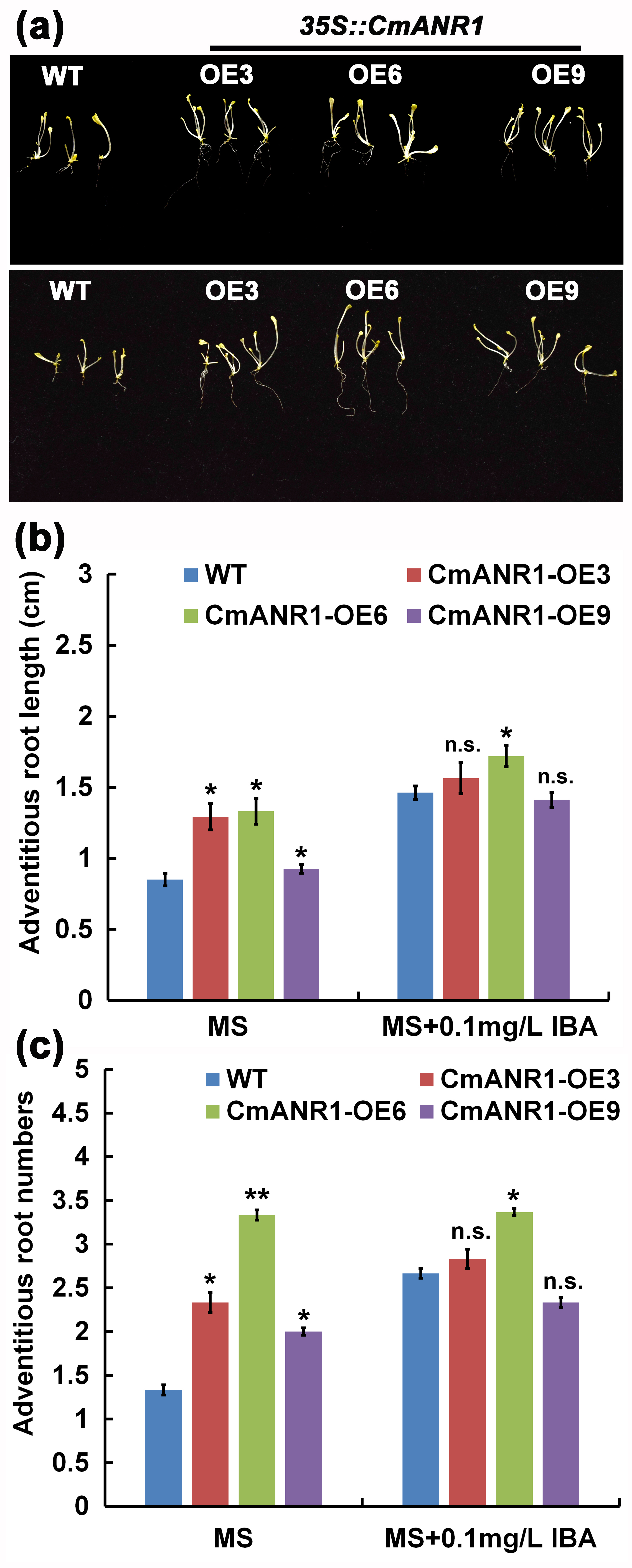


**Supplementary Figure S2. The AR rooting on hypocotyls of *CmANR1*-transgenic (OEs) and wild-type (WT) *Arabidopsis* under dark condition.** (**a**) The phenotypes of AR in *CmANR1*-transgentic and WT *Arabidopsis* on MS and MS+IBA medium. (**b**) Average length of AR in OEs and WT *Arabidopsis* on MS and MS+IBA medium. (**c**) The number of AR in OEs and WT *Arabidopsis* on MS and MS+IBA medium. Data are shown as the mean ± SE and the number of observations in each mean is 15. Statistical significance was determined using a Student's *t* test. n.s: *P* > 0.01; *: *P* < 0.01; **: *P* < 0.001.


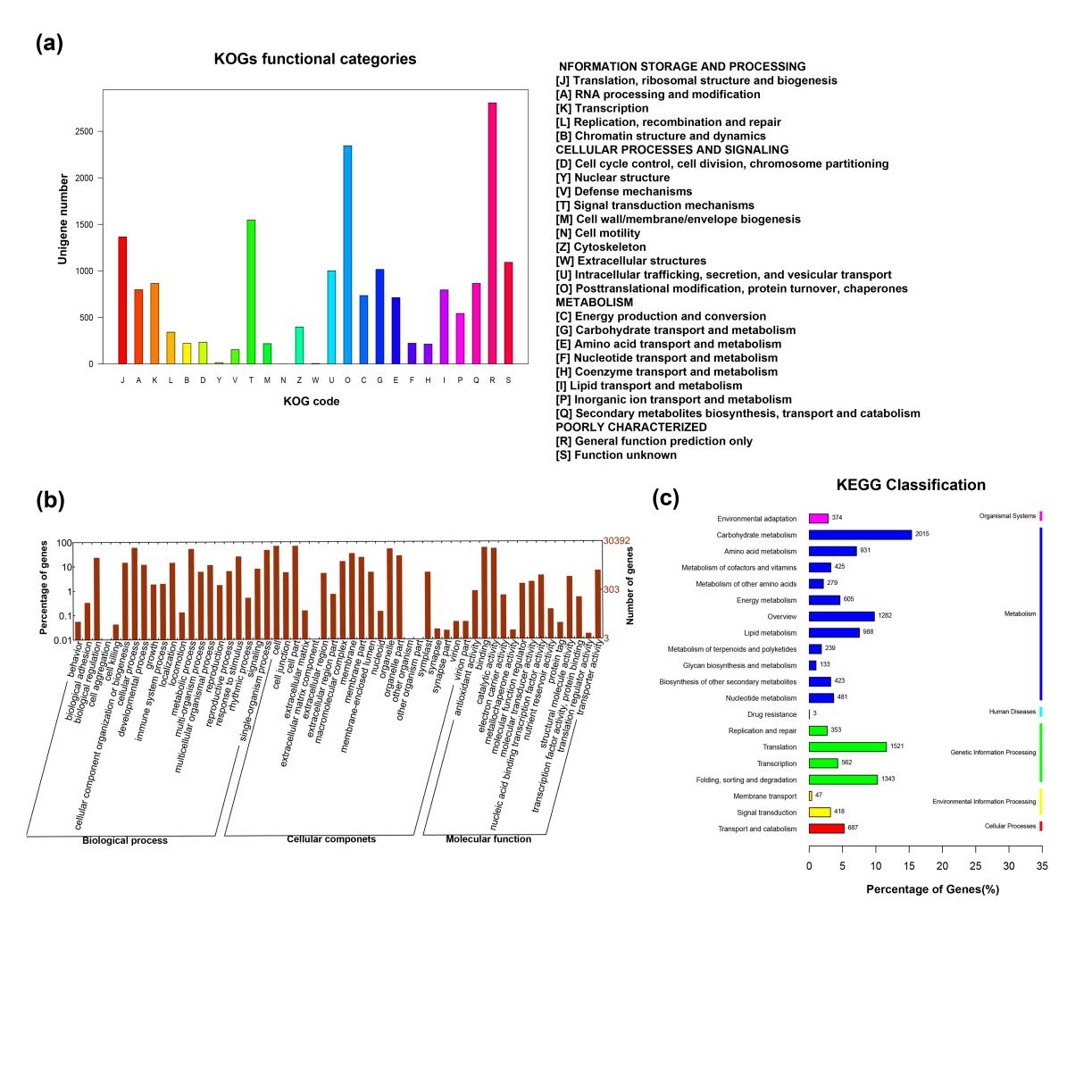


**Supplementary Figure S3. Transcriptome sequencing and annotation of the high-quality assembled unigenes in roots of chrysanthemum.** (**a**) KOG functional categories of the high-quality assembled unigenes. The detailed explanation of the code are listed on the right column. (**b**) GO annotation classification of these unigenes. The X-axis represents functional terms, while the two Y-axises mean percent and numbers of the unigenes in three categories. (**c**) KEGG pathway classification of these unigenes. The numbers represent the number of unigenes annotated in the corresponding pathways, while the percent is listed in the Y-axis. Different colors means different pathways, while the same color indicates the same category.


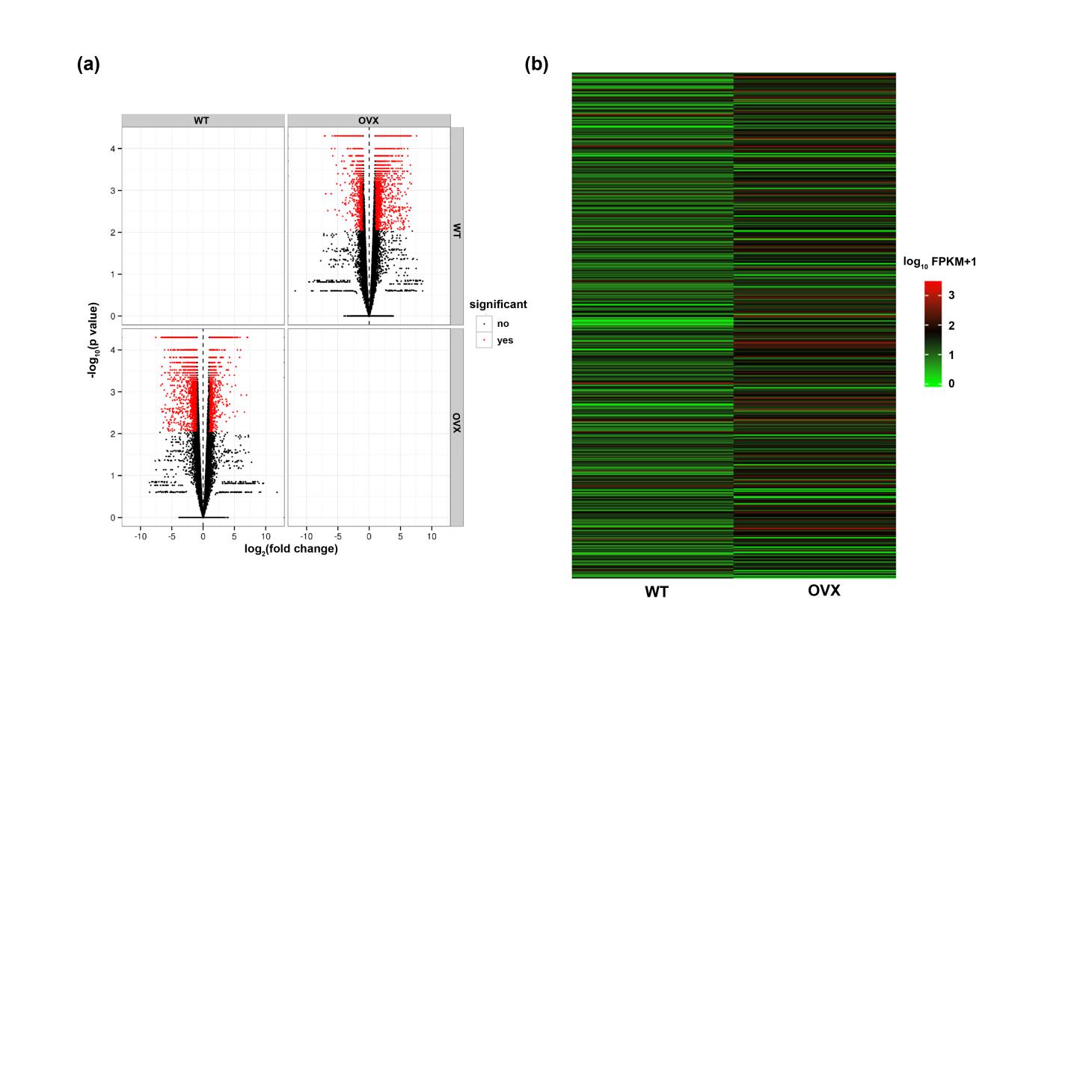


**Supplementary Figure S4.** **Expression profiles of differently expressed genes (DEGs).** (**a**) MA Value Plot of the DEGs in WT and OVXs. One small dot in the picture means a unigene, the X-axis represents A-value, the log Kow of the average FPKM [Log_10_ (FPKM)] between WT and OVX; the Y-axis is equal to M-value, the log Kow of the fold change of the expression differences, Log_2_ (Fold Change). Unigene with significant expression difference is highlighted in red dot. (**b**) Cluster Digram of DEGs between WT and OVXs. All DEGs are listed in the Y-axis, while the samples of WT and OVXs are indicated on X-axis. The degree of color represents the expression level of the DEGs: the red color signifies high expression level, the green color indicates low expression level.

**
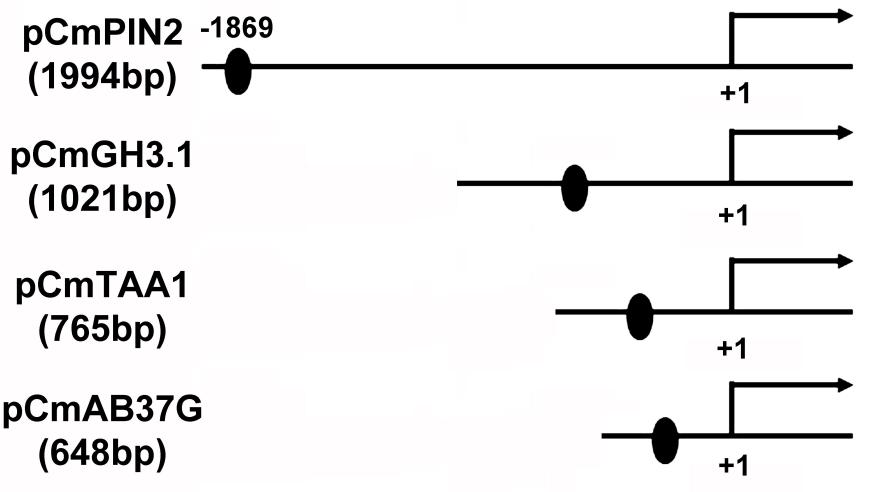
**

**Supplementary Figure S5.** **Schematic diagram of CArG-box motifs in the promoters of auxin-responsive genes including *CmPIN2*, *CmGH3.1*, *CmTAA1* and *CmAB37G*.**

**Supplementary Table S1.** Phenotype of *CmANR1-*transgenic and WT chrysanthemum (tissue culture).

| Sample name | Number of ARs | Total Length  (cm) | Ave Length  (cm) | SurfArea (cm2) | RootVolume  (cm3) |
| --- | --- | --- | --- | --- | --- |
| WT | 8.7±1.2^C^ | 40.1±1.6^C^ | 4.55±0.71^C^ | 9.6±0.4^C^ | 2.8±0.1^C^ |
| OVX56 | 15.5±1.3^A^ | 96.5±2.1^A^ | 6.22±1.09^A^ | 17.2±1.4^A^ | 6.1±0.3^A^ |
| OVX67 | 17.3±1.0^A^ | 114.1±2.8^A^ | 6.57±0.87^A^ | 18.1±0.8^A^ | 7.4±0.1^A^ |
| OVX81 | 12.5±0.6^B^ | 64.9±0.5^B^ | 5.16±0.53^B^ | 12.5±0.3^B^ | 3.9±0.2^B^ |

Numbers are presented as mean ± SE. The number of observations in each mean is nine. Means in the same column followed by the same letter are not significantly different (P < 0.05, LSD test). Date was recorded from 20 day-old tissue-cultured plants.

| Sample name | Number of ARs | Number of LRs | Total Length  (cm) | SurfArea  (cm2) | Root Volume  (cm3) | Shoot Height  (cm) |
| --- | --- | --- | --- | --- | --- | --- |
| WT | 20.5±2.1^C^ | 194.7±13.3^C^ | 372.3±9.7^C^ | 54.9±3.1^C^ | 7.3±0.4^C^ | 16.1±0.5^C^ |
| OVX56 | 37.2±1.4^A^ | 328.6±4.2^B^ | 790.9±10.3^A^ | 137.5±5.4^A^ | 18.5±0.2^A^ | 22.6±0.8^A^ |
| OVX67 | 38.0±1.7^A^ | 400.5±11.4^A^ | 837.8±14.1^A^ | 150.5±4.3^A^ | 21.2±0.3^A^ | 24.4±1.5^A^ |
| OVX81 | 26.3±2.7^B^ | 229.3±13.2^C^ | 550.8±13.4^B^ | 96.3±3.0^B^ | 11.8±0.6^B^ | 18.7±0.9^B^ |

**Supplementary Table S2.** Phenotype of *CmANR1*-transgenic and WT chrysanthemum (hydroponic culture).

Numbers are presented as mean ± SE. The number of observations in each mean is nine. Means in the same column followed by the same letter are not significantly different (P < 0.05, LSD test). Date was recorded from 40 day-old hydroponic-cultured plants.

**Supplementary Table S3.** *De novo* transcription group assembly statistics.

N50 represents the length of a transcript when the cumulative bases reaches 50% of the total numbers. Unigene adopts the same method.

**Supplementary Table S4.** The primers used in this study.

| **Gene** | **Primers(5’ to 3’)** | **Description** |
| --- | --- | --- |
| TR2418\|c1_g1(AUX1) | F:ATGCTGCGGAGAAGCCACC | qRT-PCR |
|  | R:CCACCCTCCAAATCCGAAAC |  |
| TR18026\|c1_g1(PIN2) | F:AATAGCATGTGGGCAGCGAGTG |  |
|  | R:GCCGCCTGAACGATAGCAAC |  |
| TR3585\|c0_g1(GH3.1) | F:ACTTTTGAAGAACTAATGGACTATGC |  |
|  | R:GGGTCCAATGAGGCAAACTT |  |
| TR20589\|c0_g1(PIN3) | F:CCCCTCTTCAGTTTTGTGCTAT |  |
|  | R:ATCGTAAGCACCGAACACTCCT |  |
| TR21678\|c0_g10(RHM1) | F:ATCAGACCTCAACAACCCACG |  |
|  | R:CCTTTAGGTTTCTCTTTGCCATT | qRT-PCR |
| TR22489\|c0_g1(BT5) | F:CACGACAAGGTATTGAACGAGC |  |
|  | R:GAGTTGCCACATCCGTTTGC |  |
| TR21070\|c0_g1(AB37G) | F:CACCACACAAACAAAGAGAAAACAG |  |
|  | R:GCATTTCCTGTGAAGGCTCG |  |
| TR21843\|c0_g1(TAA1) | F:GAAGCCCCATTTTGCTGACTAT |  |
|  | R:CACTAACAACCATCCTCTTCACCC |  |
| TR18879\|c1_g1(ILR1)-F | F:GGTGACAATGGGAGGTGAAGAT |  |
|  | R:AATAAGGCGAGTGCAAATGCT |  |
| TR10188\|c0_g1(CML16) | F:AACTAACTGAAATGATGCGTGAAGC |  |
|  | R:TCCTAAGCCGCCGTGACCCT |  |
| TR13908\|c0_g2(CML50) | F:AGGATGGGTTCGGTAAAGGG |  |
|  | R:CGAGTTGTTATTTTAGCCAAGTGG |  |
| TR10111\|c1_g1(CDPK33) | F:TGGTAGCGGGTTTATTACAAGAGAC |  |
|  | R:TTCCCGTCGTTATCGGTGTC |  |
| TR1588\|c0_g19(PBP1) | F:AGAAGAATTCATCAGTTTTGGGTC |  |
|  | R:ATCAAATTCGGACTTAATCTAAACAT |  |
| TR6635\|c0_g1(CIPK6) | F:GGTTATGATGGTGCCAAAGCG |  |
|  | R:GCCTCGGGAGATCTTTTTATACATG |  |
| TR15148\|c0_g1(CYCD3;2) | F:TCGGAGTTGGTCAGGAGGTG | qRT-PCR |
|  | R:GATAATCGTATGCATTGTCAGGATC |  |
| TR2164\|c0_g1(CYCB1;4) | F:ATTTTGTCGAGTATTGGAGGTGTG |  |
|  | R:GGGTTCGCCGTTTACCTCTT |  |
| TR20380\|c0_g1(ACS7) | F:AGGGAAAGATTGCGGAAACG |  |
|  | R:CCCAAATTCATCCAACAAAACAAC |  |
| TR21309\|c3_g14(ERF109) | F:AGCGGCTATTGAGTTTAGAGGACC |  |
|  | R:GCCACAGGCTGACTATTTTCG |  |
| CmUBI | F:CTAATGAATGCTTACTGTGACCGAC |  |
|  | R:AGGCGAATCATCAGTACCAAGTG |  |
| CmANR1(qPCR) | F:ATGGGGAGAGGTAAGATCGTGAT |  |
|  | R:GTGCTAGAGAAGATCATAACACC |  |
| CmPIN2(SP1) | CAAAGTATCAGCCGCAATAAACCTA | Promoter cloning |
| CmPIN2(SP2) | AACAAAGCGATTTATCCCGGAACAC |  |
| CmPIN2(SP3) | CGTAGAGTGGAACCATTGCGGACAT |  |
| CmPIN2(SP4) | ATTGTGGCATTGTGTGCAATAAGC |  |
| CmPIN2(SP5) | GGTTACTACACTACCGCCACTACACG |  |
| CmPIN2(SP6) | GAAGGATTTAGTGGTTGGTTTTGTGAC |  |
| pPIN2 | F:AAATGCGGATGCTAATGATTGGAG |  |
|  | R:TTTTGGAAGGTTTTTGTTTTGGGG |  |
| pPIN2-Pro(EMSA) | F:TTAAAAGGCCTAAATG**CATTATAAAG**TAGTATAGATAATTAG | EMSA |
|  | R:CTAATTATCTATACTA**CTTTATAATG**CATTTAGGCCTTTTAA |  |
| pPIN2-mPr(EMSA) | F:TTAAAAGGCCTACATG**TTCCTTGGGA**TAGTATAGATAATTAG |  |
|  | R:CTAATTATCTATACTA**TCCCAAGGAA**CATGTAGGCCTTTTAA |  |
| pPIN2(CHIP) | F:TAAGTCTTCTAATTTTAAAAGGCCT | ChIP-qPCR |
|  | R:GAAACAAACTCAACAGCATTACC |  |
| pTAA1(CHIP) | F:TGTCACAGTGCTTGTATTCAGTCCT | ChIP-qPCR |
|  | R:ACATCGCGTGCATTATTTCATT |  |
| pAB37G(CHIP) | F:CGGGTCAGGTAATGGGTCAA |  |
|  | R:ACCTGTTTGTTACCTTTGGTTTTAG |  |
| pCmGH3.1(CHIP) | F:TCATACACCCATTTATATTTCAGCA |  |
|  | R:CTCTGCCTCGTCGGTTTTATCT |  |
| CmPIN2pro::Luc | F(PstI):CTGCAGAAATGCGGATGCTAATGATTGGAG | Luc imaging |
|  | R(BamHI):GGATCCTTTTGGAAGGTTTTTGTTTTGGGG |  |
| CmANR1(35Spro) | F(SmaI):CCCGGGATGGGGAGAGGTAAGATCGTG |  |
|  | R(SalI):GTCGACATGTAGTTGTAATCCCAAATTTGTAG |  |
| CmANR1-His | F(BamHI):GGATCCATGGGAAGAGGGAAGATTGTGAT | CmANR1-  fusion protein |
|  | R(XhoI):CTCGAGTTCGTTTGCTCTTGGTGGAGTG |  |
